# Estimation of duplication history under a stochastic model for tandem repeats

**DOI:** 10.1186/s12859-019-2603-1

**Published:** 2019-02-06

**Authors:** Farzad Farnoud, Moshe Schwartz, Jehoshua Bruck

**Affiliations:** 1 Department of Electrical and Computer Engineering, Department of Computer Science, University of Virginia, Charlottesville, USA; 2 Department of Electrical and Computer Engineering, Ben-Gurion University of the Negev, Beer Sheva, Israel; 3 Department of Electrical Engineering, California Institute of Technology, Pasadena, USA

**Keywords:** Tandem repeats, Duplication history, Stochastic approximation, Estimation

## Abstract

**Background:**

Tandem repeat sequences are common in the genomes of many organisms and are known to cause important phenomena such as gene silencing and rapid morphological changes. Due to the presence of multiple copies of the same pattern in tandem repeats and their high variability, they contain a wealth of information about the mutations that have led to their formation. The ability to extract this information can enhance our understanding of evolutionary mechanisms.

**Results:**

We present a stochastic model for the formation of tandem repeats via tandem duplication and substitution mutations. Based on the analysis of this model, we develop a method for estimating the relative mutation rates of duplications and substitutions, as well as the total number of mutations, in the history of a tandem repeat sequence. We validate our estimation method via Monte Carlo simulation and show that it outperforms the state-of-the-art algorithm for discovering the duplication history. We also apply our method to tandem repeat sequences in the human genome, where it demonstrates the different behaviors of micro- and mini-satellites and can be used to compare mutation rates across chromosomes. It is observed that chromosomes that exhibit the highest mutation activity in tandem repeat regions are the same as those thought to have the highest overall mutation rates. However, unlike previous works that rely on comparing human and chimpanzee genomes to measure mutation rates, the proposed method allows us to find chromosomes with the highest mutation activity based on a single genome, in essence by comparing (approximate) copies of the pattern in tandem repeats.

**Conclusion:**

The prevalence of tandem repeats in most organisms and the efficiency of the proposed method enable studying various aspects of the formation of tandem repeats and the surrounding sequences in a wide range of settings.

**Availability:**

The implementation of the estimation method is available at http://ips.lab.virginia.edu/smtr.

**Electronic supplementary material:**

The online version of this article (10.1186/s12859-019-2603-1) contains supplementary material, which is available to authorized users.

## Background

Tandem repeats, which form about 3% of the human genome [1], are segments of DNA that primarily consist of repeats of a certain pattern. The number of copies in tandem repeats is highly variable and is prone to change due to tandem duplication mutations. Furthermore, tandem repeats are subject to point mutations [2]. The variability of tandem repeats enables them to be used for population genetics [3] and forensics [4]. Tandem repeats may cause expansion diseases, gene silencing [5], and rapid morphological variation [6].

A mechanisms suggested for the formation of tandem repeat sequences, especially those of shorter lengths, is slipped-strand mispairing [7], also known as replication slippage [8]. This mechanism refers to the misalignment of the template and the nascent strand during DNA replication. It is thought that the presence of near-identical sequences increases the probability of misalignment [7].

In this work, we present and analyze a model of the evolution of tandem repeat sequences via tandem duplication and substitution mutations. The starting point is a short sequence which we refer to as the *seed*. At each mutation step, either a tandem duplication or a substitution mutation occurs, each with a given probability. Here, a tandem duplication refers to a type of duplication in which a newly created copy of a segment of the sequence (the template) is inserted into the same sequence immediately after the template. Thus, the model is appropriate for studying slippage-driven repeats but not designed to represent repeats resulting from other processes, such as recombination [9]. The length of the seed, also referred to as pattern length, may be from one to hundreds of nucleotides. However, generally only repeats with short pattern lengths, e.g. 1–10 nt, are associated with polymerase slippage [7]. In the model, tandem duplications of different lengths do not necessarily have the same probability. We show analytically that certain statistical features of the sequence converge as the number of mutations increases. This in turn allows us to i) predict the behavior of the sequence after a large number of mutations if we have the parameters of the model, or ii) estimate the parameters of the model given the sequence after a large number of mutations. In other words, given a sequence that is the result of the aforementioned process, we can estimate conditional mutation probabilities without any other information or comparison with homologous sequences from other organisms.

We study two cases in the evolution of tandem repeats. First, we consider the case in which substitution mutations do not occur and the only type of mutation is tandem duplication. We show that in this case, while the prediction of evolutionary behavior is easy, estimation of model parameters, including the probabilities of tandem duplications of given lengths, is difficult. This is because as the number of mutations increases, the sequence demonstrates periodic behavior, lacking features that can be leveraged for estimation. Perhaps surprisingly, the period of this sequence is not necessarily the most common or the shortest possible tandem duplication length.

We then consider the more interesting case in which both tandem duplication and substitution mutations occur. In this case, substitutions disrupt the periodic pattern that would arise from tandem duplications. As a result, after a large number of mutations, the resulting sequence is more complex and informative, allowing us to estimate the model parameters. Specifically, from such a sequence, we can estimate the probability of a substitution in each step, as well as the probabilities of tandem duplications of different lengths. Furthermore, we can estimate the total number of mutations that gave rise to the sequence under study. We apply this method to the tandem repeats in the human genome, which enables us to investigate the prevalence of substitutions in repeats of different lengths and to compare the average number of mutations among chromosomes. We show that two classes of tandem repeats are observed based on their mutation profiles and that this classification is compatible with the mini- and micro-satellite classification based on pattern length. Furthermore, our analysis illustrates that the average number of mutations in some chromosomes are higher than others. Interestingly, this agrees with another measure of mutation activity, i.e., comparison with the chimpanzee genome: The chromosomes with higher mutation counts in repeated regions are the same as the ones that have diverged most from chimpanzee chromosomes.

Our results demonstrate that the proposed estimation method can be used to study various aspects of tandem repeat sequences, such as the effects of different factors on mutation rates, at a large scale. Such studies will be helpful for understanding what factors affect the occurrences of diseases that result from tandem repeats, such as repeat expansion diseases [5]. More accurate estimates of the number of mutations will also enable a better characterization of the relationship between cancer and repeat instability [10]. Classification of tandem repeats based on mutation profile is informative for understanding the differences between the underlying mutation mechanisms. Furthermore, such a classification will lead to more accurate choices of distance metrics between sequences with similar mutation profiles, based on how likely each mutation type is. These metrics can then be used to obtain improved phylogenetic trees using tandem repeat sequences.

### Related work

This paper presents an explicit stochastic model for the evolution of tandem repeat sequences. Conventional models of sequence evolution, such as those of Jukes and Cantor [11], Kimura [12], and Felsenstein [13], focus on substitution mutations and are not applicable to more complex mutations such as insertions, deletions, and duplications. While the study of more complex models has proved challenging [14], they have been studied by some authors, including [14–16] for deletions and insertions, and [17–19] for tandem duplications.

In previous work on modeling tandem duplication and substitution mutations, it is often assumed that in each step, the length of the sequence grows by at most one repeat unit, which simplifies the analysis; see, e.g., [18] and references therein. Our model however allows duplications of lengths longer than one repeat unit at a time. Note that models that do not allow longer duplications may underestimate the probability of substitution and overestimate the probability of tandem duplication since more duplication events are needed to account for the observed copy number. Models proposed by [17, 19] include duplications of lengths longer than one repeat unit. But these works only consider perfect tandem repeats, in which all copies are identical. Imperfect tandem repeats, however, are common in genomic data. Furthermore, unlike [17, 18] that use Markov chains and branching processes for modeling, our analysis is based on stochastic approximation, which enables the description of new aspects of the problem. In particular, we see that the observed period in a tandem repeat sequence is not necessarily the most common duplication length (Theorem 1) and that the presence of substitutions allows the estimation of mutation probabilities (9). Finally, these papers are not concerned with recovering the duplication history, which is a focus of the current paper. Stochastic analysis has also been used by [20, 21], to study latent periodicity in genomic sequences. There, the goal is to utilize statistical analysis to improve upon the purely spectral methods of period detection for both genomic and non-genomic data, rather than the estimation of the duplication history.

Recovering the duplication history has been studied by [22–24], which take a combinatorial approach to solving the problem. Via simulation, we show that the method proposed in this paper outperforms the state-of-the-art method, called DTSCORE [24]. Estimation of the duplication history using a stochastic model, to the best of our knowledge, has not appeared in the literature before.

## Modeling and estimation method

We will first present an overview of our method. Our approach relies on designing a stochastic model for the evolution of tandem repeats in the presence of tandem duplication and substitution mutations. Assuming the parameters of the model (the conditional probabilities of duplication and substitution mutations) are known, we study the asymptotic behavior of tandem repeat sequences. This analysis is based on the autocorrelation function since this feature well represents the (approximate) periodicity that results from duplication and substitution mutations. We determine the limit set of the autocorrelation function as a function of model parameters. We will then address the inverse problem of estimating the parameters given a sequence, assuming that its autocorrelation is close to the limit. This in turn enables us to estimate the counts of mutations of different types in the history of the sequence.

### Model and general analysis via stochastic approximation

In this section, we first present the stochastic model and a general framework for analyzing the evolution of sequences under duplication and substitution mutations using stochastic approximation. Stochastic approximation relates the behavior of a discrete system to an ordinary differential equation (ODE) [25], which is often more tractable. The use of stochastic approximation for the analysis of stochastic mutation models was originally proposed by [26] to study the evolution of the frequencies of *k*-mers in a simplified model of interspersed duplication. After setting up the model and the preliminaries, we study the behavior of the autocorrelation function in systems with tandem duplication and substitution.

Let *s* be a circular sequence over some alphabet $\mathcal A$ that “evolves” over time. The process starts with *s*^(0)^, called the *seed*, and in each step, *s*^(*i*)^ is obtained from *s*^(*i*−1)^ through a random mutation. The reason that we choose *s* to be a circular string, and not a linear one, is to avoid the technical difficulties of dealing with its boundaries. If the mutation occurring at time *i* is a substitution, its position is chosen at random among all symbols of *s*^(*i*)^. That symbol is then changed randomly to one of the other symbols of $\mathcal {A}$. If the mutation is a tandem duplication of length *ℓ*, a substring of length *ℓ* is chosen uniformly at random, duplicated, and inserted in tandem. We use *q*_0_ to denote the probability that the mutation in any given step is a substitution and *q*_*ℓ*_, *ℓ*>0, to denote that it is a tandem duplication of length *ℓ*. We assume that there exists *K* such that *q*_*ℓ*_=0 for all *ℓ*>*K*. Finally, we let **q**=(*q*_0_,*q*_1_,…,*q*_*K*_) where $\sum _{i=0}^{K}q_{i}=1$. Note that **q** represents conditional mutation probabilities given that a mutation occurs and not the mutation probabilities per generation. In our notation *s*^(*i*)^ is the instance of *s* at time *i*. However, if it causes no ambiguity, we may use *s* instead of *s*^(*i*)^. We use *L*_*i*_ to denote the length of *s*^(*i*)^.

For an ordered set *U*, let $\boldsymbol {R}_{n}=\left (R_{n}^{u}\right)_{u\in U}$ be a vector representing the number of appearances of objects *u*∈*U* in the sequence *s* at time *n* and let $\boldsymbol {\rho }_{n}=\frac {\boldsymbol {R}_{n}}{L_{n}}$ be the normalized version of ***R***_*n*_. For example, *U* can be the set of all strings over $\mathcal {A}$ with length at most three. Our goal is to find out how ***ρ***_*n*_ changes with *n* by finding a differential equation whose solution approximates ***ρ***_*n*_.

Define $\mathcal {F}_{n}$ to be the filtration generated by the random variables {***ρ***_*n*_,*L*_*n*_}. Furthermore, let $\mathbb {E}_{\ell }\left [ \cdot \right ]$ denote the expected value conditioned on the fact that the length of the duplicated substring is *ℓ* and let $\boldsymbol {\delta }_{\ell }=\mathbb {E}_{\ell }\left [\boldsymbol {R}_{n+1}|\mathcal {F}_{n}\right ]-\boldsymbol {R}_{n}$. Recall that *q*_0_ is the probability of a substitution and *q*_*i*_,0<*i*≤*K* is the probability of the event that a sequence of length *ℓ*=*i* is duplicated.

To understand how ***ρ***_*n*_ varies, our starting point is the difference sequence ***ρ***_*n*+1_−***ρ***_*n*_. Similar to [26] and as described in the Additional file 1 for completeness, it can be shown that

1 $$ \boldsymbol{\rho}_{n+1}-\boldsymbol{\rho}_{n}=\frac{1}{L_{n}}\left(\boldsymbol{h} (\boldsymbol{\rho}_{n})+\boldsymbol{M}_{n+1}+O\left(L_{n}^{-1}\right)\right),  $$

where ***h***_*ℓ*_(***ρ***)=***δ***_*ℓ*_(***ρ***)−*ℓ****ρ*** and $\boldsymbol {h}(\boldsymbol {\rho })={\sum }_{\ell =0}^{K}q_{\ell }\boldsymbol {h}_{\ell }(\boldsymbol {\rho })$, and where $\boldsymbol {M}_{n+1}=\boldsymbol {R}_{n+1}-\mathbb {E}\left [\boldsymbol {R}_{n+1}|\mathcal {F}_{n}\right ]$ is a bounded martingale difference sequence.

This system can be analyzed through stochastic approximation ([25], Theorem 2), by relating the discrete system describing ***ρ***_*n*_ to a continuous system. In particular, the sequence ***ρ***_*n*_ converges almost surely to a compact connected internally chain transitive invariant set of the ODE

2 $$ \frac{d\boldsymbol{\rho}_{t}}{dt}=\boldsymbol{h}\left(\boldsymbol{\rho}_{t}\right).  $$

While different properties of the sequence can be analyzed via the aforementioned method, for our purpose, the autocorrelation of the sequence is the most suitable, as it captures the degree of repetitiveness of sequences arising from tandem duplication. The autocorrelation function *R*^*r*^ of a sequence $s=s_{1}\dotsm s_{\left |s\right |},\,s_{i}\in \mathcal {A}$, at lag *r*, is defined as

$$R^{r}=\sum\limits_{i=1}^{\left|s\right|}\langle s_{i}, s_{i+r} \rangle, $$

where indices of *s* are computed modulo |*s*| and 〈*α*,*β*〉=1 if *α*=*β* and 〈*α*,*β*〉=0 otherwise.

Let $R_{n}^{r}$ denote the autocorrelation of function after *n* mutations starting from the seed sequence and let $\rho _{n}^{r}=\frac {R_{n}^{r}}{L_{n}}$. To express autocorrelation as a vector, let $\boldsymbol {R}_{n}=\left (R_{n}^{0},R_{n}^{1},\dotsc,R_{n}^{m-1}\right)$ and $\boldsymbol {\rho }_{n}=\frac {\boldsymbol {R}_{n}}{L_{n}}$, for a constant *m*. Note that $R_{n}^{0}=L_{n}$ and $\rho _{n}^{0}=1$.

To find the ODE of Eq. (2), we need to find $\boldsymbol {h}_{\ell }(\boldsymbol {\rho })= \left ({h}_{\ell }^{0}(\boldsymbol {\rho }),\dotsc,{h}_{\ell }^{m-1}(\boldsymbol {\rho })\right)$. As shown in Additional file 1,

3 $$ h_{\ell}^{r}(\boldsymbol{\rho})=\left\{ \begin{array}{ll} -\frac{8}{3}{\rho}^{r}+\frac{2}{3}, & \qquad \ell=0\\ r{\rho}^{r-\ell}-r{\rho}^{r}, & \qquad \ell>0 \end{array}\right.   $$

From Eq. (2), we have

4 $$ \frac{d}{dt}{\rho}_{t}^{r} =q_{0}\left(-\frac{8}{3}{\rho}_{t}^{r} + \frac{2}{3}\right)+r\sum_{\ell>0}q_{\ell}{\rho}_{t}^{r-\ell}-\left(1-q_{0}\right)r{\rho}_{t}^{r}  $$

for 0<*r*≤*m*−1. We thus see that the set of equations governing ***ρ*** are linear.

For *m*≥*K*, we can write Eq. (4) as

5 $$ \frac{d}{dt}\boldsymbol{\rho}_{t}=A\boldsymbol{\rho}_{t},  $$

where *A* is the *m*×*m* matrix whose rows and columns are indexed by {0,1,…,*m*−1} and its elements are given as

6 $$ A_{rj}=\left\{ \begin{array}{ll} 2q_{0}/3+rq_{r}, & \qquad\text{if \(r>j=0\),}\\ rq_{r-j}+rq_{r+j}, & \qquad\text{if \(r>j>0\),}\\ q_{0}\left(r-\frac{8}{3}\right)+rq_{2r}-r, & \qquad\text{if \(r=j>0\),}\\ rq_{r+j}, & \qquad\text{if \(j>r>0\),}\\ 0, &\qquad\text{\(r=0\).} \end{array}\right.   $$

As discussed in Additional file 1, ***ρ***_*t*_ converges to some ***ρ***_*∞*_ satisfying

7 $$ A\boldsymbol{\rho}_{\infty}=0,  $$

It can then be shown that ***ρ***_*n*_ converges almost surely to the null space of *A*.

In the following sections, we consider the null space of *A* in two cases. First, we assume *q*_0_=0, that is, there are no substitutions. Next we study the case with positive probability of substitutions, i.e., *q*_0_>0.

### Tandem duplication

In this section, we consider the case in which the only type of occurring mutations is tandem duplication. We show that in this case the null space of *A* is simple.

#### **Theorem 1**

Suppose *q*_0_=0. Let *P*={*i*:*i*>0,*q*_*i*_>0} and $d=\gcd P$. The normalized autocorrelation $\boldsymbol {\rho }_{n}=\left (\rho _{n}^{0},\dotsc,\rho _{n}^{m-1}\right)$ converges almost surely to a vector $\boldsymbol {\rho }_{\infty }=\left (\rho _{\infty }^{0},\dotsc,\rho _{\infty }^{m-1}\right)$, where ${\rho }_{\infty }^{j}$ is periodic in *j* with period *d*, ${\rho }_{\infty }^{j}=1$ if *j*≡0 (mod *d*), and ${\rho }_{\infty }^{j}={\rho }_{\infty }^{d-j}$. In particular, every pair of symbols at distance *d* in *s*^(*n*)^ are, with high probability, the same.

The theorem implies that regardless of the seed, after many duplications, the sequence becomes almost periodic with period *d*. The periodicity is expected since no substitutions occur. However, the period is not the dominant or the shortest duplication length, but rather it is the gcd of all lengths *i* for which the probability of duplication *q*_*i*_ is positive. For example, if duplications of lengths 4 and 6 occur, the sequence becomes approximately periodic with period 2. Since given *P*, *d* does not depend on the values of the *q*_*i*_, observing *d* does not provide enough information for estimating **q** and thus, in this case, we are not able to solve the inverse problem. Nevertheless, the study of this case lays the foundation for the more complex case in which substitutions are present and where we are able to solve the inverse problem.

To prove Theorem 1, we need the following lemma whose proof is given in Additional file 1.

#### **Lemma 1**

Let *q*_0_=0, *P*={*i*>0:*q*_*i*_>0}, and $d=\gcd P$. Furthermore, let *S*(*t*)=Span{***v***_0_,…,***v***_⌊*t*/2⌋_}, where ***v***_*i*_=(*v*_*i*,0_,…,*v*_*i*,*m*−1_)^*T*^, with

$$v_{i,j}=\left\{ \begin{array}{ll} 1, & \quad j\equiv\pm i\pmod{t},\\ 0, & \quad\text{otherwise.} \end{array}\right. $$

We have Null (*A*)=*S*(*d*).

#### *Proof of Theorem 1:*

Since ***ρ***_*∞*_ is in the null space of *A*, where the null space of *A* is given by Lemma 1, ***ρ***_*∞*_ is a linear combination of the vectors *S*(*d*). Furthermore, by definition we know that $\rho _{\infty }^{0}=1$. In the basis of *S*(*d*) given in Lemma 1, the only vector that has a nonzero element in the 0th coordinate is ***v***_0_. So the coefficient of ***v***_0_ in the linear combination describing ***ρ***_*∞*_ is 1 and thus $\rho _{\infty }^{j}=1$ if *j*≡0 (mod *d*). We hence have Theorem 1. □

### Tandem duplication and substitution

We now consider both tandem duplication and substitution mutations and describe how the parameters of the model, as well as the number of mutations of each type, may be estimated. Note that while the parameters of the model are unknown, we have access to the sequence *s*^(*n*)^ for some *n*.

The following lemma (see Additional file 1 for proof) states that the autocorrelation function converges to a single point when both duplication and substitution mutations are present. This fact will facilitate the design of the estimator.

#### **Lemma 2**

Let *q*_0_>0, *P*={*i*>0:*q*_*i*_>0}, $d=\gcd P$, and let *A* be the matrix of Eq. (6). We have Null (*A*)=Span(***v***), where ***v***=(*v*_0_,…,*v*_*m*−1_)^*T*^ is a vector satisfying *v*_0_=1 and $v_{j}=\frac {1}{4}$ for *j*≢0 (mod *d*).

For example, for $d\,=\,3, \boldsymbol {v}\,=\,\left (1,\frac {1}{4}, \frac {1}{4},v_{3}, \frac {1}{4},\frac {1}{4},v_{6},\frac {1}{4},\dotsc \right)^{T}.$

From the lemma, it follows that there is only one valid solution to the equation *A****ρ***_*∞*_=0 which satisfies ${\rho }_{\infty }^{0}=1$. This unique point is the limit of the autocorrelation function.

We have thus shown that if we know **q**, we can determine ***ρ***_*∞*_. We now turn to the estimation problem, which is the inverse of determining ***ρ***_*∞*_ using **q**. In other words, we are given a sequence whose autocorrelation we can compute and our goal is to determine **q**.

Note that we can rewrite the equation *A****ρ***_*∞*_=0, where *A* is the matrix given in Eq. (6), as

8 $$ C \mathbf{q}=\tilde{\boldsymbol{\rho}_{\infty}},  $$

where **q** = (*q*_0_,*q*_1_,…,*q*_*m*_)^*T*^ and $\tilde {\boldsymbol {\rho }_{\infty }}=\left ({\rho }_{\infty }^{1}, 2 {\rho }_{\infty }^{2},\dotsc, (m-1){\rho }_{\infty }^{m-1}\right)^{T}$, and where *C*=(*C*_*ri*_) is a (*m*−1)×(*m*+1) matrix whose elements are

$$C_{ri}= \left\{ \begin{array}{ll} \frac{2}{3}+\left(r-\frac{8}{3}\right){\rho}_{\infty}^{r}, & \qquad i=0\\ r{\rho}_{\infty}^{i-r}, & \qquad\text{otherwise,} \end{array}\right. $$

where *r*∈{1,…,*m*−1} and *i*∈{0,1,…,*m*}.

Given ***ρ***_*∞*_, we can solve Eq. (8) for **q**. Since we only know the sequence after a finite time *n*, we approximate ***ρ***_*∞*_ by $\boldsymbol {\rho }_{n} =\left (\rho _{n}^{0},\dotsc,\rho _{n}^{m-1}\right)$ computed from *s*^(*n*)^. In our model, there exists *K* such that *q*_*i*_=0 for *i*>*K*. However, the value of *K* is unknown to us. We thus choose some *m*^′^ and assume that *q*_*i*_=0 for *i*>*m*^′^. The value of *m*^′^ can be chosen for example based on our knowledge of the underlying biological processes, such as slipped-strand mispairings [7], that lead to tandem repeats. Furthermore, the value of *m*^′^ should be chosen large enough so that *m*^′^≥*K* with a high degree of confidence. Note that there are *m*^′^+1 unknown quantities, namely, the elements $q_{0},\dotsc,q_{m^{\prime }}$ of **q**. Another parameter is the number of equations used to estimate **q**, denoted *m*^′′^, which should be chosen close to *m*^′^. Having chosen *m*^′^,*m*^′′^, we can write Eq. (8) as $C^{\prime } \mathbf {q}=\tilde {\boldsymbol {\rho }_{n}}$, where $\mathbf {q}=\left (q_{0},q_{1},\dotsc,q_{m^{\prime }}\right)^{T}$ and $\tilde {\boldsymbol {\rho }_{n}}=\left ({\rho }_{n}^{1},2{\rho }_{n}^{2},\dotsc,m^{\prime \prime }{\rho }_{n}^{m^{\prime \prime }}\right)^{T}$, and where *C*^′^ is the matrix containing the first *m*^′′^ rows and the first *m*^′^+1 columns of *C*, computed using ***ρ***_*n*_ instead of ***ρ***_*∞*_. Now to obtain an estimate of **q** we can solve the least-square curve fitting problem

9 $$ \begin{aligned} \hat{\mathbf{q}}=\arg\min_{\mathbf{q}}\ & \left\Vert C^{\prime}\mathbf{q}-\tilde{\boldsymbol{\rho}_{n}}\right\Vert_{2}^{2}\\ \text{s.t.} \,& \mathbf{q}^{T}\mathbf{1}=1\\ & q_{i}\ge0,\text{ for }0\le i \leq m^{\prime}. \end{aligned}  $$

The solution $\hat {\mathbf {q}}$ of this problem contains an estimate of the substitution probability *q*_0_ and the probabilities *q*_*ℓ*_ of duplications of lengths *ℓ*. Noting that the expected length added to the sequence by each mutation is ${\sum }_{i=1}^{m^{\prime }}i\hat {q}_{i}$, we estimate the total number *n* of mutations that have occurred as

10 $$ \hat{n}=\frac{\left|s^{(n)}\right|-\left|s^{(0)}\right|}{{\sum}_{i=1}^{m^{\prime}}i\hat{q}_{i}},  $$

where we assume the length of the seed *s*^(0)^ is equal to the pattern length. The estimator based on the proposed Stochastic Model of Tandem Repeats and defined by Eqs. (9) and (10) is referred to as SMTR.

In tandem repeat sequences observed in genomes duplication events have lengths that are multiples of a certain value, leading to a pattern of that length appearing many times. We refer to this length as the *pattern length* and to the number of times that the pattern appears as the *copy number*. While in general SMTR does not need to know the pattern length *d*, if it is known, we set *q*_*i*_=0 for *i*≢0(mod d). Furthermore, from Lemma 2, we know ${\rho }_{\infty }^{r}=1/4$ for *r*≢0(mod d). Replacing these values in Eq. (10) allows us to solve it by keeping only rows and columns of *C*^′^ whose indices are multiples of *d*.

We note that in Eq. (10), if $\hat {q}_{0}$ is close to 1, then the estimate $\hat {n}$ for *n* may be very large. It is reasonable to expect that $\hat {n}$ is not larger than the length of the sequence. Thus, we add the constraint $\left (d,2d,\dotsc,m^{\prime } d\right)\left (q_{d},q_{2d},\dotsc,q_{m^{\prime } d}\right)^{T}\ge 1$ to Eq. (9), where *d* is the pattern length. This ensures that on average each mutation contributes at least 1 to the length of the sequence. Furthermore, since our method relies on asymptotic approximation, for short sequences, specifically those with copy number ≤3, we provide an alternative heuristic estimation algorithm, which is described in Additional file 1.

## Simulation and data analysis results

In this section, we use simulation to evaluate the performance of SMTR by comparing its estimates of the model parameters with the true values. We also compare SMTR to DTSCORE introduced by [27], which was shown to outperform similar methods [24]. Further, we apply SMTR to tandem repeats in the human genome to study variation across chromosomes and pattern lengths.

In the results that follow, we set the computation parameters as follows. First, we find ***ρ***=(*ρ*^*r*^) for $r=0,1,\dotsc,\left \lfloor \frac {|s|}{2}\right \rfloor $. This ensures that each value of the autocorrelation function is the average of at least |*s*|/2 values. Furthermore, we let $m^{\prime }=m^{\prime \prime }=\min \left (\max (10d,5r^{*}),\left \lfloor \frac {|s|}{2}\right \rfloor \right)$, where *r*^∗^= arg max*rρ*^*r*^. The max here is intended to ensure that *m*^′^ is large enough, while the min ensures that all needed values of *ρ* are available. Finally, while the estimation method is geared towards tandem repeats with substitution mutations, our inspection of the results shows that for perfect tandem repeats, the algorithm returns probability near zero for substitution mutations, as expected, and nearly uniform probability for different duplication lengths. Thus, in the results that follow, we apply it to tandem repeats regardless of the apparent presence of substitution mutations.

### Simulation results

We now turn to evaluating the performance of SMTR through simulation and also compare it with DTSCORE [27]. We show that SMTR provides more accurate estimates and is significantly faster compared to DTSCORE.

In our simulation set up, we first generate a random seed *s*^(0)^ of a random length *d* that then undergoes *n* random substitutions and tandem duplications, where the probabilities of these events are given by **q**, itself randomly generated. The resulting sequence *s*^(*n*)^ and the pattern length *d* are then passed to the SMTR estimator, which of course does not know *s*^(0)^, *n*, or **q**. We evaluate the performance by finding the *L*_2_ error in estimating $\hat {\mathbf {q}}$, $\|\hat {\mathbf {q}}-{\mathbf {q}}\|_{2}$, averaged across *N* experiments for each value of *n*. We also find the normalized root mean square (NRMS) error in estimating *n*. For a given value of *n*, NRMS Error is defined as

$$\text{NRMSE}(n)=\frac{1}{n}\sqrt{\frac{1}{N}\sum\limits_{i=1}^{N}\left(\hat{n}_{i}-n\right)^{2}}\, $$

where *N* is the number of experiments with *n* mutations and $\hat {n}_{i}$ is the estimate for *n* in the *i*th experiment.

We find the errors for two different cases: for a pair of given values for *n* and **q**, we estimate $\hat {n}$ and $\hat {\mathbf {q}}$ based on 1) a single sequence and 2) *n*_*s*_ sequences all generated with parameters **q** and *n*. In the latter case, estimates are obtained for each sequence individually and then averaged. The multiple-sample case is intended to show that performance improves, as expected, with more data. Due to the large number of tandem repeat sequences in many genomes, it is reasonable to expect that for a set of factors affecting duplication probabilities, e.g., GC content and pattern length, a given set of values for these factors is likely to arise multiple times. When studying the effects of such factors on mutation rates, we may expect a similar performance improvement by averaging the estimates among all instances with the same set of values for the factors.

**Fig. 1:**
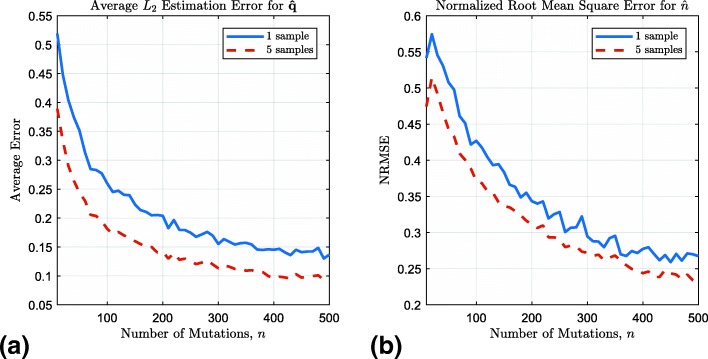
Errors of the estimate $\hat {\mathbf {q}}$ of **q**, (**a**), and the estimate $\hat {n}$ of *n*, (**b**)

More detail on the simulation setup is given in Additional file 1. The results are given in Fig. 1 where *n* ranges from 10 to 500, with step size equal to 10. For each value of *n*, the experiment is performed *N*=500 times, and in each of these *N* trials, estimates are obtained based on a single sequence and based on *n*_*s*_=5 sequences drawn for the same seed and **q**. We observe that as *n* increases, the errors sharply decrease. For a single sequence and a small number of mutations, the estimation algorithm relies on a very limited amount of data. As the number *n* of mutations increases, the sequence becomes longer, providing more data in the form of the autocorrelation function and asymptotic approximations become more accurate. It is also observed that with more samples for the same set of parameters, more accurate estimates are obtained.

**Fig. 2:**
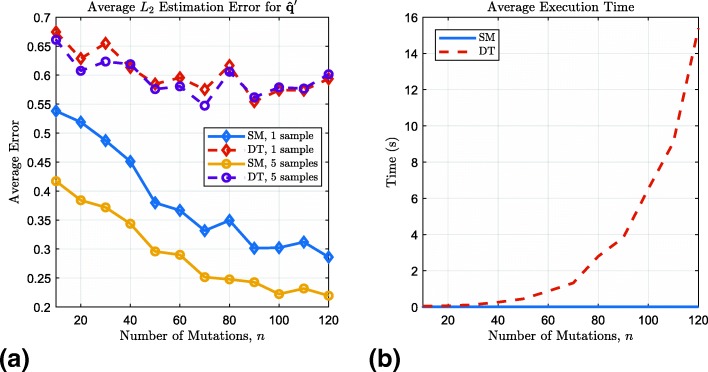
Comparison of SMTR Estimation (SM) and DTSCORE (DT): Error of $\hat {\mathbf {q}}^{\prime }$, (**a**), and the average execution time for an instance of the problem on an Intel Core i7–7700 CPU with 16 GB of RAM, (**b**)

We now compare the performance of SMTR with DTSCORE [27]. DTSCORE is a distance-based algorithm designed to find the duplication history in the form of a tree, thus providing estimates for the counts of duplications of various lengths. In [24], it was shown that DTSCORE performs better than other algorithms for identifying the duplication tree, including TRHIST [22] and WINDOWS [23]. Due to the slower speed of DTSCORE (the worst-case time complexity is *O*(*L*^4^), where *L* is the copy number), we restrict the range of the number of mutations *n* to {10,20,…,120} and also reduce *N*=200 but maintain *n*_*s*_=5. As the distance measure, we use Jukes-Cantor’s [11], which is compatible with our sequence generation method. The comparison is given in Figure 2. Since from DTSCORE, we can only derive estimates for the counts of duplications but not substitutions, we compare the accuracy of estimating $\mathbf {q}^{\prime }=\left (q^{\prime }_{1},q^{\prime }_{2},\dotsc \right)$ where $q^{\prime }_{i}$ for *i*≥1 is defined as $q^{\prime }_{i}=\frac {q_{i}}{1-q_{0}}\ \cdot $

From Fig. 2a, it is clear that SMTR estimates **q**^′^ with significantly higher accuracy than DTSCORE. Furthermore, if multiple samples from the same distribution are available, the improvement for SMTR is larger than for DTSCORE. Finally, the execution time of SMTR is faster than DTSCORE. In particular, for *n*=120, on average, SMTR needs no more than 0.015 s to compute the estimate for each tandem repeat sequence, while DTSCORE needs 15 s, 3 orders of magnitude longer. As a result, SMTR will scale better when analyzing a large number of tandem repeats, for example, all repeats in a given chromosome or genome.

While we have shown the improved accuracy and efficiency of SMTR compared to DTSCORE, we note that combinatorial methods such as DTSCORE are more generic in the sense that they do not rely on a stochastic model of the generation of tandem repeats. On the other hand, DTSCORE is more restrictive in the sense that it assumes duplications occur at the predefined boundaries of tandem repeat blocks (copies of the pattern). Blocks are meaningful if each copy is a gene, but in general, they are logical constructs rather than biological entities. Finally, it is worth noting that both DTSCORE and SMTR are designed for the analysis of repeats resulting from polymerase slippage and not recombination events.

### Tandem repeats in the human genome

We now apply SMTR to tandem repeats in the human genome to estimate the number of substitution and tandem duplication mutations for each. We use these estimates to explore the variation of mutation rates for minisatellite and microsatellites and across chromosomes. Most of the results provided in this section rely on estimating the number of substitutions in tandem repeat sequences. We note that the DTSCORE algorithm only provides estimates for duplication events. Furthermore, due to its efficiency, SMTR is more appropriate for large-scale data analysis.

We use the Tandem Repeats Database (TRDB) [28], which provides the set of tandem repeats in each chromosome, as identified by the Tandem Repeat Finder (TRF) algorithm, and related information such as the length of the repeat unit and indel (insertion/deletion) percentage. As a preprocessing step, among overlapping repeats, we keep only one. We also remove repeats with unknown (N) bases and those with copy number less than 2. Finally, we discard repeats whose indel percentage is nonzero, as our model does not include insertion and deletion mutations. We note however that the indel percentage is an approximate value for the number of apparent insertions and deletions. Excluding repeats with non-zero indel percentages does not guarantee that there will be no insertions or deletions in the remaining repeats. Another limitation is that our method assumes substitutions are unbiased, and so it cannot take into account different transition and transversion probabilities, or the effect of GC content. As an example of the preprocessing step, the number of repeat sequences in chromosome 1 reduces from 93,626 to 38,628 as a result of preprocessing.

**Fig. 3:**
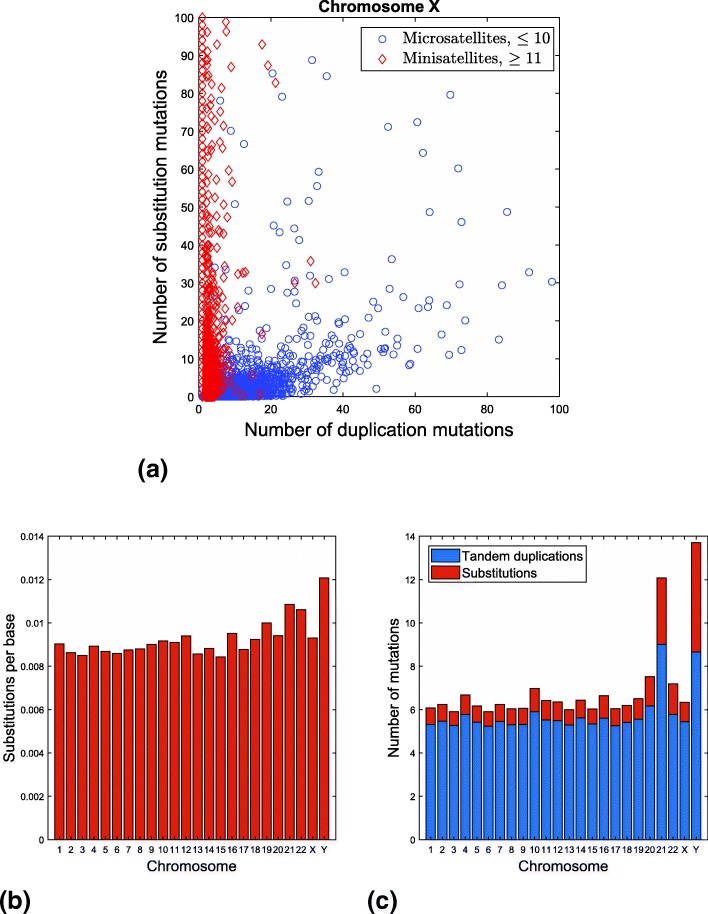
The mutation profile of tandem repeats in chromosome X (**a**) and mutation variation across chromosomes in microsatellites (pattern length ≤10): Mean of the ratio of the number of substitutions to the length of the tandem repeat sequence (**b**) and mean of the total number of mutations per tandem repeat sequence for each chromosome (**c**)

We applied the SMTR algorithm to tandem repeats in each chromosome to study the role of tandem duplication and substitution mutations in their formation. The results for chromosome X are given in Fig. 3a. Each point in this plot corresponds to a tandem repeat sequence. The position of each point is determined by the estimated number of tandem duplications and substitutions that occurred to create the sequence. It can be observed that tandem repeat sequences can roughly be divided into two clusters with different behaviors: one dominated by tandem duplication mutations and the other by substitution mutations. This difference in behavior matches well with the classification of tandem repeats as microsatellites and minisatellites, with pattern lengths of 1–10 and 11–100 bases, respectively. Other chromosomes exhibit behavior similar to chromosome X illustrated here. Among all chromosomes, the minimum Kendall tau correlation coefficient between the rankings of repeats based on length of the pattern and based on the fraction of mutations that are substitutions was 0.5160. Given the large number of tandem repeats in each chromosome, such high correlation coefficients lead to p-values that are practically zero (as computed with MATLAB).

We now turn our attention to evaluating the variation of mutation rates across chromosomes. Through comparison with the chimpanzee genome [29–31], it is known that mutation rates vary across chromosomes. To see whether this variation can also be observed in repeated regions, we study the number of mutations in tandem repeat sequences across chromosomes. Since our model represents replication slippage, we only consider tandem repeats with short patterns. Specifically, for tandem repeats with pattern length ≤10, we estimate the number of substitution and duplication mutations. As a measure of mutation activity, we find the average of the ratio of the the number of substitutions to the length of the tandem repeat sequence for each chromosome (Fig. 3b). The top five chromosomes that have the highest substitution rates are Y, 21, 22, 19, and 16. Based on comparison with the chimpanzee genome [31], the five chromosomes with highest mutation activity are Y, 21, 19, 22, and 16. Thus the top five chromosomes are the same based on the two approaches (*p*-value=0.00002). We repeated this analysis for repeats with maximum pattern lengths of 8, 9, 11, and 12, and in all cases, at least four of the top five matched the result from comparison with chimpanzee [31].

We also considered the average number of mutations per tandem repeat for each chromosome (Fig. 3c). On average, tandem repeats in chromosome 21 have a higher number of mutations than other autosomes. The average number of duplication mutations is estimated to be higher in chromosome 21 than in the Y chromosome. The higher number of mutations in chromosome 21 compared to other autosomes is also observed if we set the upper bound on the length of the patterns at 8, 9, 11, and 12.

## Discussion

Figure 1 demonstrates that compared to DTSCORE, the proposed method, SMTR, is both more accurate and faster. The efficiency of SMTR allows it to be applied at the genome scale. Such large-scale analyses enable statistically studying hypotheses about the formation of tandem repeats.

We studied the relationship between the length of the pattern in a tandem repeat and number of substitution and duplication mutations. A clear difference emerges between minisatellites and microsatellites, as shown in Fig. 3a. The different mutation profiles suggest that these two types of tandem repeats may result from different mutation mechanisms. This is compatible with previous findings, where polymerase slippage is thought to give rise to microsatellites while unequal recombination is believed to cause the heterogeneity observed in minisatellites [32]. Our method is only designed to model slippage and not recombination. The fact that it generally estimates the number of substitutions to be higher for minisatellites than microsatellites can be the result of higher raw heterogeneity that is observed in microsatellites and/or caused by model mismatch. The results of this analysis suggests that it is possible to design statistical tests to decide the origin of tandem repeat sequences, as a means of classifying them, rather than relying on classification merely based on pattern length.

Figure 3b presents the normalized number of substitutions in tandem repeats, averaged for each chromosome. As discussed, the five chromosomes with the highest rates in Fig. 3b are the same as the five chromosomes with the highest mutation rates, as obtained by [31] based on comparison with chimpanzee genome. This suggests a strong relationship between substitutions in repeated regions and overall mutation activity in chromosomes. On the other hand, the results are not exactly aligned. For example, while chromosome X has the smallest divergence from chimpanzee, it does not have the smallest normalized number of substitutions. Overall, our results suggest estimation of mutation activity based on tandem repeats can be a powerful tool in studying mutations since unlike existing methods it relies on a single genome rather than on comparison of genomes from different species.

In Fig. 3c, the reason that tandem repeats in chromosome 21 exhibit a higher number of mutations is unknown to us but it is interesting to note that individuals with trisomy 21 can survive into adulthood, which suggests that mutations in chromosome 21 are relatively better tolerated. It is also observed that 3 of the 5 chromosomes with the highest total number of mutations in microsatellites, Y, 21, and 22, match the result from [31]. This further suggests a higher mutation activity in these chromosomes. However, care should be taken in interpreting results about mutation counts that are not normalized by the length of the sequence. The opportunity for mutation increases with length and copy number. In particular, increased copy number may increase the probability of misalignment during replication [33]. Another factor that can affect the number of mutations in a complex manner is the interplay between substitution mutations and tandem duplication mutations: if many substitutions occur, the copies become more heterogeneous, which may decrease the possibility of misalignment. This interaction is not taken into account in our model and left to future work.

## Conclusion

In this paper, we introduced a new stochastic model for tandem duplication and substitution mutations, and analyzed it via stochastic approximation. In particular, we fully characterized the limit set of the stochastic process described by the model. In addition to enabling us to predict the behavior of a sequence that undergoes tandem duplication and substitution mutations, this characterization allowed us to derive a minimization problem whose solutions are estimates of the conditional mutation probabilities for tandem duplication and substitution. We showed further that it is possible to estimate the total number of mutations. Finally, we evaluated the estimation method via simulation by generating random sequences and comparing the estimated probabilities with the true values and also applied it to the human genome, where it demonstrated the differing behavior of micro- and mini-satellites as well as the variability of mutation activity across chromosomes.

Advantages of our method include its scalability and the fact that it relies on a single sequence to infer occurrences of mutations. While with this method, we can learn only about mutations in tandem repeat regions, our results show that the findings may be applicable to surrounding regions and can be of use in forming hypotheses about mutation activity, for example, about factors that increase or decrease activity.

There still exist many open problems in stochastic modeling and estimation for tandem repeats. For example, the model presented here does not take into account deletions nor the fact that the level of heterogeneity may affect the probability of tandem duplication. Neither does the model consider bias in substitution mutations. For example, it cannot reflect different transversion and transition probabilities. Incorporating such biases will make the method more appropriate, for instance, for GC rich repeats. Further, we only analyzed it in the asymptotic regime and left finite-time behavior to future work. Finite-time analysis will enable us to analytically quantify the accuracy of the presented estimation method as a function of the number *n* of mutations and to devise improved estimation algorithms. Finally, further work is needed to accurately model mutations other than DNA slippage that cause duplication, especially those that lead to minisatellite repeats.

## Additional file

Additional file 1 Supplementary Material. Section 1 (§SM.1) Proof of (1). §SM.2: Proof of (3). §SM.3: Proof of (7). §SM.4: Proof of Lemma 1. §SM.5: Proof of Lemma 2. §SM.6: Estimation for copy number ≤3. §SM.7: Simulation Setup. (PDF 244 kb)

## Abbreviations

ODE: Ordinary differential equation
NRMSE: Normalized root mean square error
