# Supplementary material for: Estimation of duplication history under a stochastic model for tandem repeats
Source: BMC Bioinformatics. 2019 Feb 6;20:64. doi: 10.1186/s12859-019-2603-1 (PMC6364452; doi:10.1186/s12859-019-2603-1)
Supplement: Supplementary file 1 — Supplementary Material. Section 1 (§SM.1) Proof of (1). §SM.2: Proof of (3). §SM.3: Proof of (7). §SM.4: Proof of Lemma 1. §SM.5: Proof of Lemma 2. §SM.6: Estimation for copy number ≤3. §SM.7: Simulation Setup. (PDF 244 kb) [file 12859_2019_2603_MOESM1_ESM.pdf]

Estimation of Duplication History under a Stochastic Model for Tandem Repeats Supplementary Material

Farzad Farnoud (Hassanzadeh), Moshe Schwartz, Jehoshua Bruck

SM.1 Proof of Eq. (1)

The following set of conditions must be satisfied for our analysis. Among them, we assume (A1). It is easy to see that (A2)-(A3) are true, and (A4)-(A5) will be evident after we find $\boldsymbol{\delta}_\ell = \mathbb{E}_\ell\left[\boldsymbol{R}_{n+1}|\mathcal{F}_n\right] - \boldsymbol{R}_n$.

(A1) There exists $K \in \mathbb{N}$ such that $q_i = 0$ for $i > K$.

(A2) $\boldsymbol{R}_{n+1} - \boldsymbol{R}_n$ is bounded and, thus, so is $\boldsymbol{\delta}_\ell$.

(A3) $\boldsymbol{\rho}_n$ is bounded.

(A4) For each ℓ, $\boldsymbol{\delta}_\ell$ is a function of $\boldsymbol{\rho}_n$ only (so we can write $\boldsymbol{\delta}_\ell = \boldsymbol{\delta}_\ell\left(\boldsymbol{\rho}_n\right)$).

(A5) The function $\boldsymbol{\delta}_\ell\left(\boldsymbol{\rho}_n\right)$ is Lipschitz.

Note that

$$\boldsymbol{\rho}_{n+1} - \boldsymbol{\rho}_n = \mathbb{E}\left[\boldsymbol{\rho}_{n+1} - \boldsymbol{\rho}_n|\mathcal{F}_n\right] + \left(\boldsymbol{\rho}_{n+1} - \mathbb{E}\left[\boldsymbol{\rho}_{n+1}|\mathcal{F}_n\right]\right). \tag{S.1}$$

For the first term of the right side of Eq. (S.1), we have

$$\begin{aligned}\mathbb{E}\left[\boldsymbol{\rho}_{n+1}-\boldsymbol{\rho}_n\middle|\mathcal{F}_n\right] &= \sum_{\ell=0}^{K} q_\ell\left(\mathbb{E}_\ell\left[\boldsymbol{\rho}_{n+1}\middle|\mathcal{F}_n\right]-\boldsymbol{\rho}_n\right)\\ &= \sum_{\ell=0}^{K} q_\ell\left(\frac{\boldsymbol{R}_n+\boldsymbol{\delta}_\ell\left(\boldsymbol{\rho}_n\right)}{L_n+\ell}-\frac{\boldsymbol{R}_n}{L_n}\right)\\ &= \frac{1}{L_n}\sum_{\ell=0}^{K} q_\ell\boldsymbol{h}_\ell\left(\boldsymbol{\rho}_n\right)\left(1+O\left(L_n^{-1}\right)\right)\\ &= \frac{1}{L_n}\boldsymbol{h}\left(\boldsymbol{\rho}_n\right)\left(1+O\left(L_n^{-1}\right)\right), \qquad \text{(S.2)}\end{aligned}$$

where $\boldsymbol{h}_\ell(\boldsymbol{\rho})=\boldsymbol{\delta}_\ell(\boldsymbol{\rho})-\ell\boldsymbol{\rho}$, $\boldsymbol{h}(\boldsymbol{\rho})=\sum_{\ell=0}^{K}q_\ell\boldsymbol{h}_\ell(\boldsymbol{\rho})$, and where we have used $1/(L_n+\ell)=(1+O(L_n^{-1}))/L_n$ which follows from the boundedness of ℓ (see (A1)).

Furthermore, for the second term of the right side of Eq. (S.1), we have

$$\begin{aligned}\boldsymbol{\rho}_{n+1}-\mathbb{E}\left[\boldsymbol{\rho}_{n+1}\middle|\mathcal{F}_n\right] &= \frac{\boldsymbol{R}_{n+1}}{L_{n+1}}-\mathbb{E}\left[\frac{\boldsymbol{R}_{n+1}}{L_{n+1}}\middle|\mathcal{F}_n\right]\\ &= \frac{1+O\left(L_n^{-1}\right)}{L_n}\left(\boldsymbol{R}_{n+1}-\mathbb{E}\left[\boldsymbol{R}_{n+1}|\mathcal{F}_n\right]\right)\\ &= \frac{1}{L_n}\left(1+O\left(L_n^{-1}\right)\right)\boldsymbol{M}_{n+1} \qquad \text{(S.3)}\end{aligned}$$

where $\boldsymbol{M}_{n+1}=\boldsymbol{R}_{n+1}-E\left[\boldsymbol{R}_{n+1}|\mathcal{F}_n\right]$. Note that $\boldsymbol{M}_n$ is a bounded martingale difference sequence.

From Eq. (S.1), Eq. (S.2), and Eq. (S.3), we find

$$\boldsymbol{\rho}_{n+1}=\boldsymbol{\rho}_n+\frac{1}{L_n}\left(\boldsymbol{h}\left(\boldsymbol{\rho}_n\right)+\boldsymbol{M}_{n+1}+O\left(L_n^{-1}\right)\right),$$

where we have used the fact that $\boldsymbol{h}(\boldsymbol{\rho}_n)(1+O(L_n^{-1}))=\boldsymbol{h}(\boldsymbol{\rho}_n)+O(L_n^{-1})$. This follows from the boundedness of $\boldsymbol{h}(\boldsymbol{\rho}_n)$, which in turn follows from the boundedness of $\boldsymbol{\delta}(\boldsymbol{\rho}_n)$.

SM.2 Proof of Eq. (3)

Let $s = s^{(n)} = s_1 \cdots s_{|s|}$ be the sequence at time n. At time $n+1$, either a substitution or a duplication has happened. In the former case, suppose the symbol at position i is changed to another symbol of the alphabet, and in the latter case, suppose that the substring $s_{i+1} \cdots s_{i+\ell}$ is duplicated in a tandem manner; after duplication the sequence becomes

$$s_1 \cdots s_i s_{i+1} \cdots s_{i+\ell} s_{i+1} \cdots s_{i+\ell} s_{i+\ell+1} \cdots s_{|s|}.$$

Fix the value of i. For $\ell = 0$, i.e., the case of a substitution,

$$R_{n+1}^r = R_n^r - \langle s_i, s_{i+r} \rangle - \langle s_i, s_{i-r} \rangle + \langle s_i', s_{i+r} \rangle + \langle s_i', s_{i-r} \rangle , \tag{S.4}$$

where s_i' denote the new (mutated) symbol.

Now we consider the case of $\ell > 0$, which corresponds to tandem duplications. For $0 < \ell \leq r$, we have

$$R_{n+1}^r = R_n^r - \sum_{j=i+\ell-r+1}^{i} \langle s_j, s_{j+r} \rangle + \sum_{j=i+\ell-r+1}^{i+\ell} \langle s_j, s_{j+r-\ell} \rangle . \tag{S.5}$$

The conditions resulting in the first summation $j \leq i$ and $j + r > i + \ell$ and those resulting in the second summation are $i < j \leq i + \ell$ or $i + \ell < j + r \leq i + 2\ell$. For $\ell > r$,

$$R_{n+1}^r = R_n^r + \sum_{j=i+1}^{i+\ell-r} \langle s_j, s_{j+r} \rangle + \sum_{j=i+\ell-r+1}^{i+\ell} \langle s_j, s_{j+r-\ell} \rangle . \tag{S.6}$$

Note that $\boldsymbol{h}_\ell(\boldsymbol{\rho}_n) = \left(h_\ell^0(\boldsymbol{\rho}_n), \ldots, h_\ell^{m-1}(\boldsymbol{\rho}_n)\right) = \boldsymbol{\delta}_\ell - \ell\boldsymbol{\rho}_n$. To compute $\boldsymbol{\delta}_\ell = \mathbb{E}_\ell[\boldsymbol{R}_{n+1}|\mathcal{F}_n] - \boldsymbol{R}_n$, we first find the following expected values, where $r > 0$ and where i is randomly and

uniformly distributed among the L_n positions:

$$
\begin{aligned}
\mathbb{E}_0\left[\langle s_i, s_{i+r}\rangle | \mathcal{F}_n\right] &= \mathbb{E}_0\left[\langle s_i, s_{i-r}\rangle | \mathcal{F}_n\right] = \rho_n^r, \\
\mathbb{E}_0\left[\langle s_i', s_{i+r}\rangle | \mathcal{F}_n\right] &= \mathbb{E}_0\left[\langle s_i', s_{i-r}\rangle | \mathcal{F}_n\right] = \frac{1-\rho_n^r}{3}, \\
\mathbb{E}_\ell\left[\sum_{j=i+\ell-r+1}^{i} \langle s_j, s_{j+r}\rangle \middle| \mathcal{F}_n\right] &= \frac{1}{L_n}\sum_{i=1}^{L_n}\sum_{j=i+\ell-r+1}^{i} \langle s_j, s_{j+r}\rangle \\
&= (r-\ell)\,\rho_n^r, \\
\mathbb{E}_\ell\left[\sum_{j=i+\ell-r+1}^{i+\ell} \langle s_j, s_{j+r-\ell}\rangle \middle| \mathcal{F}_n\right] &= \frac{1}{L_n}\sum_{i=1}^{L_n}\sum_{j=i+\ell-r+1}^{i+\ell} \langle s_j, s_{j+r-\ell}\rangle \\
&= r\rho_n^{r-\ell}, \\
\mathbb{E}_\ell\left[\sum_{j=i+1}^{i+\ell-r} \langle s_j, s_{j+r}\rangle \middle| \mathcal{F}_n\right] &= \frac{1}{L_n}\sum_{i=1}^{L_n}\sum_{j=i+1}^{i+\ell-r} \langle s_j, s_{j+r}\rangle \\
&= (\ell-r)\,\rho_n^r.
\end{aligned}
$$

Using Eq. (S.4), Eq. (S.5), and Eq. (S.6), we can find $\boldsymbol{\delta}_\ell$ and thus $\boldsymbol{h}_\ell$. It can also be verified that (A4)-(A5) hold. Eq. (3) then follows.

SM.3 Proof of Eq. (7)

To determine the stability of Eq. (5) we use the Gershgorin circle theorem. We note that $\sum_j A_{rj} = -2q_0$ and that $A_{rr} = -\frac{8q_0}{3} - r\,(1 - q_0 - q_{2r})$ for $r > 0$. The circles centered at A_{rr} and with radius $\sum_j A_{rj} - A_{rr} = \frac{2q_0}{3} + r\,(1 - q_0 - q_{2r})$ in the complex plane either do not intersect the right half of the plane or they intersect it only at 0. Hence, by the Gershgorin circle theorem, the eigenvalues of A are either 0 or have negative real parts. Let $(\lambda_j)_{j=0}^{m-1}$ denote the eigenvalues of A, with $\lambda_0 = 0$ and $\lambda_j = a_j + \imath b_j$ for $j > 0$, where $a_j < 0$ and $\imath$

denotes $\sqrt{-1}$. For $0 \le r \le m-1$, we have

$$\rho_t^r = c_{r0}(t) + \sum_{j=1}^{m-1} c_{jk}(t)\, e^{a_j t + \imath b_j t},$$

where $c_{rj}(t)$ are polynomials in t of degree at most m. Since $\rho^r(t)$ is bounded between 0 and 1, it is evident that $c_{r0}(t)$ is in fact a constant. Let this constant be denoted by ρ_∞^r. We thus have

$$\rho_t^r = \rho_\infty^r + \sum_{j=1}^{m-1} c_{jk}(t)\, e^{a_j t + \imath b_j t}, \tag{S.7}$$

which implies that $\left(\rho_t^0, \ldots, \rho_t^{m-1}\right)$ converges to $\boldsymbol{\rho}_\infty = (\rho_\infty^0, \ldots, \rho_\infty^{m-1})$. Note that $\rho_\infty^0 = 1$.

From Eq. (S.7), we have $\lim_{t\to\infty} \frac{d}{dt}\rho_t^r = 0$. By taking the limit of the equation $\frac{d}{dt}\boldsymbol{\rho}_t = A\boldsymbol{\rho}_t$ as $t \to \infty$, it follows that

$$A\boldsymbol{\rho}_\infty = 0,$$

implying that $\boldsymbol{\rho}_\infty$ is in the null space of A.

SM.4 Proof of Lemma 2

Proof. Let $B = (B_{rj})$ be an $m \times m$ matrix, with rows and columns indexed by $0, \ldots, m-1$, defined by

$$B_{rj} = \begin{cases} q_r, & \text{if } j = 0 \\ q_{r-j} + q_{r+j}, & \text{if } 0 < j < r \\ q_{2r} - 1, & \text{if } j = r \\ q_{r+j}, & \text{if } j > r \end{cases} \tag{S.8}$$

Since $q_0 = 0$, we have $\mathrm{Null}(B) = \mathrm{Null}(A)$. We further recall that $q_i \in \mathbb{R}$, $q_i \in [0,1]$, and $\sum_i q_i = 1$. Additionally, assume $i_1 < i_2 < \cdots < i_k$ are the only indices for which $q_{i_j} > 0$. Finally, we assume m is large enough to enable us to see all the nonzero q_i's in the matrix, or more formally, we require $m \ge i_k$.

We are interested in finding the null-space of B. Instead of doing this directly, we consider the matrix

$$A' = I + B.$$

The goal now is to find the right eigenspace of A' for the eigenvalue 1.

First we prove $S(d) \subseteq \mathrm{Null}(B)$. We do this by showing that for all $\boldsymbol{v}_i \in S(d)$, $\boldsymbol{v}_i$ is in the right eigenspace of A' corresponding to the eigenvalue 1, i.e., $A'\boldsymbol{v}_i = \boldsymbol{v}_i$. This is immediate when we note that when $i \equiv \pm a \pmod{d}$, in the ith row of A' (numbering of rows and columns starts from 0), coordinates $j \equiv \pm a \pmod{d}$ contain all the elements q_{id}, and in particular, $q_{i_1}, q_{i_2}, \ldots, q_{i_k}$.

It is obvious that the vectors in $S(d)$ are linearly independent. To complete the proof we need to show that the geometric multiplicity of the eigenvalue 1 of A' is $|S(d)| = \lfloor d/2 \rfloor + 1$.

The matrix A' is stochastic, and therefore, its spectral radius is $\rho(A') = 1$. Let $G_{A'}$ be the (weighted) directed graph whose adjacency matrix is given by A'. By Perron-Frobenius theory, it is well known that the eigenvalues of A' are the union (in the multiset sense) of the eigenvalues of the irreducible components of $G_{A'}$. Additionally, the geometric multiplicity of $\rho(A')$ (also called the Perron-Frobenius (PF) eigenvalue of A') is 1 for each irreducible component.

Combining the above, and remembering the PF eigenvalue of an irreducible graph is a weighted average of the out-weight of its vertices, we obtain that the geometric multiplicity of $\rho(A') = 1$ is exactly the number of irreducible sink components of $G_{A'}$. Thus, as a final step in the proof, we show that the number of irreducible sink components of $G_{A'}$ is exactly $\lfloor d/2 \rfloor + 1$.

Let us denote the vertices of the graph $G_{A'}$ by $w_0, w_1, \ldots, w_{m-1}$. From each w_ℓ, $\ell > 0$, we have k out-going edges corresponding to $i_1, i_2, \ldots, i_k$. The edge corresponding to i_j is directed from w_ℓ to $w_{\ell - i_j}$ when $\ell \geq i_j$, and otherwise to $w_{i_j - \ell}$. When describing a path we shall refer to this edge as "taking i_j from w_ℓ". Finally, vertex w_0 has a single out-going edge which is also a self-loop.

By construction, all vertices w_ℓ for $\ell \geq i_k$ have incoming edges from vertices $w_{\ell'}$ with $\ell' > \ell$. Thus, they are certainly not part of an irreducible sink component. We therefore concentrate on vertices $w_0, w_1, \ldots, w_{i_k-1}$ only. We now look at the irreducible components over these vertices.

For each i_j, starting from w_ℓ, $0 < \ell < i_k$, we can take a path representing the orbit of $-i_j$ modulo i_k. If $\ell \geq i_j$, then we can move from w_ℓ to $w_{\ell-i_j}$. If $\ell < i_j$ we can also do this but we require two steps: from w_ℓ to $w_{i_j-\ell}$ by taking an i_j step, and then from $w_{i_j-\ell}$ to $w_{i_k-(i_j-\ell)}$ by taking an i_k step. Indeed

$$i_k - (i_j - \ell) \equiv \ell - i_j \pmod{i_k}.$$

Since we can do this for every i_j every node w_ℓ is connected to every node $w_{\ell'}$ with $\ell \equiv \ell'$ (mod d). Also, by taking the i_k edge from each node, we can see that from every node w_ℓ we can reach every node $w_{\ell'}$ with $\ell \equiv \pm\ell' \pmod{d}$.

The only exception to the above are nodes w_ℓ with $\ell \equiv 0 \pmod{d}$ since they get "stuck" at w_0, which is an irreducible sink component on its own. We therefore reach the conclusion that there are exactly $\lfloor d/2 \rfloor + 1$ irreducible sink components which are of the form

$$V_a = \{w_\ell \mid 0 < \ell < i_k, \ell \equiv \pm a \pmod{d}\},$$

for $0 < a \leq d/2$, as well as $V_0 = \{w_0\}$. □

SM.5 Proof of Lemma 3

Proof. Consider a matrix A' obtained from A by replacing the first all-zero row with the row vector $(1, 0, \ldots, 0)$. By a simple application of the Gershgorin circle theorem,

$$|A'_{rr}| - \sum_{j \neq r} |A'_{rj}| = 2q_0 > 0,$$

for all $r > 0$, and therefore all the eigenvalues of A' are non-zero, i.e., $\text{rank}(A') = m$. Thus, we have $\text{rank}(A) = m - 1$, and therefore $\dim \text{Null}(A) = 1$.

We now show $A\boldsymbol{v} = 0$, which along with $\dim \text{Null}(A) = 1$, implies that $\text{Null}\,(A) = \text{Span}(\boldsymbol{v})$. Let $(A\boldsymbol{v})_r$ denote the rth element of $A\boldsymbol{v}$ for $r = 0, 1, \ldots, m-1$. Since the 0th row of A is all zero, we have $(A\boldsymbol{v})_0 = 0$. Based on Eq. (6), for $(A\boldsymbol{v})_r = 0$ to hold when $r > 0$, we require

$$q_0 \left(\frac{2}{3}(1 - 4v_r) + rv_r \right) + r \sum_{k:1 \le kd \le m} v_{|kd-r|} q_{kd} - rv_r = 0.$$

This holds for $r \not\equiv 0 \pmod{d}$ if we let $v_j = \frac{1}{4}$ for all $j \not\equiv 0 \pmod{d}$. Finally, for $r \equiv 0 \pmod{d}$, $r > 0$, we can choose $v_d, v_{2d}, \ldots$ such that the above equality holds as these are not restricted in the statement of the lemma. □

SM.6 Estimation for copy number ≤ 3

Since our method relies on asymptotic approximation, for short sequences, specifically those with copy number ≤ 3, we provide an alternative estimation algorithm. In such sequences there is ≤ 2 duplication events of length equal to d and 0 or more substitutions. The number of duplications can be found easily from the length of the sequence. Let a_i be the number of distinct symbols appearing at the ith position (relative to the start of the pattern) of different copies minus 1. For example, for A̲CTG̲CTA̲CT, we have $a_1 = 1$, since two symbols, A and G, appear in the first position of different copies, and $a_2 = a_3 = 0$. The a_i can be used to infer the number of substitutions. A substitution will contribute to a_i only if it occurs after the first duplication event. To account for hidden substitutions, we estimate the number of substitutions as $(\sum_i a_i)\frac{(r+1)}{r}$, where r is the number of duplication events. So we have estimates both for the number of substitutions and the number of duplications. Note that in this simple analysis, we have assumed that each substitution results in a new symbol, which is a reasonable assumption for a small number of mutations.

SM.7 Simulation Setup

The seed sequence $s^{(0)}$ is a random sequence over the alphabet $\{\mathsf{A}, \mathsf{C}, \mathsf{G}, \mathsf{T}\}$ of a random length that is chosen uniformly from the set $\{4, 5, \ldots, 10\}$. We set d for the duplication process (all duplication lengths will be multiples of d) equal to the length of the seed. To choose $\mathbf{q}$, we choose q_0, q_d, q_{2d}, and q_{3d} by randomly selecting a point from the simplex

$$\{(\alpha_1, \alpha_2, \alpha_3, \alpha_4) \mid \alpha_1 + \cdots + \alpha_4 = 1,\ \alpha_1, \ldots, \alpha_4 \geq 0\}.$$

All other values of $\mathbf{q}$ are set to 0. We then perform n mutation steps, each a substitution with probability q_0 or a tandem duplication of length id with probability q_{id}, for $i \in \{1, 2, 3\}$. If in a tandem duplication step, the length chosen for duplication is larger than the length of the sequence, the whole sequence is duplicated. Note that since the length of the sequence grows, such an event may only happen a few times at the beginning of the process.
